# A conversation with research software engineers at the International Brain Laboratory

**DOI:** 10.1016/j.patter.2025.101315

**Published:** 2025-07-11

**Authors:** Mayo Faulkner, Miles Wells

**Affiliations:** 1 Champalimaud Foundation, Lisboa, Portugal; 2 University College London, London, UK

## Abstract

Open-source software is the lifeblood of many modern research projects, allowing researchers to push boundaries, build collaborations, and work transparently. The International Brain Laboratory (IBL), a group of more than twenty labs working together to understand the neuroscience of decision-making, uses open-source software and other open science practices extensively to advance its research. Here, we interview two of the IBL’s research software engineers to learn more about their career paths and how they view open-source development.

## Main text

### You are both now full-time software engineers, but you did not initially train in computer science. What was this transition like for you?

**Mayo Faulkner (MF):** That’s right, I only really started programming during my PhD, when I began analyzing the data I had collected. Even now, I find that having a clear, focused project is the most effective way for me to continue developing my coding skills. I’m constantly learning on the job. One of the most challenging, but also most rewarding, aspects of this work has been writing code that can scale across large and diverse datasets. It’s a continual process of finding the right balance between tailoring solutions to specific applications while keeping them general enough to be reused in other contexts.

**Miles Wells (MW):** My background is in neuroscience and, like Mayo, I began to properly learn to program when I started doing lab work. Our lab used MATLAB for all our experiments and analysis. Thankfully, one of my colleagues at the time, a PhD student, did have a background in computer science. His code was very cleverly constructed—everything generalized and following textbook design patterns, but none of it documented! I learned a lot from him, but I agree with Mayo: it’s much easier to learn a skill when there’s a tangible goal.

### What projects are you currently working on?

**MW:** Mayo and I both help manage various research projects within the International Brain Laboratory (IBL). For instance, recently I’ve spent a lot of time bringing together all of the data preprocessing for our mesoscope imaging project—a confluence of experimenter notes, histology, imaging, and behavior data—aiming to produce a dataset of neural responses simultaneously recorded across the dorsal cortex during decision-making. We also both work a lot on core infrastructure tools for ourselves and the community. Much of my work revolves around data architecture and, as our datasets grow, I’m learning new ways to manage this at scale. Before joining the IBL, I was heavily involved in animal behavior work. This still really interests me, and I’m currently working on a project to improve some of our older behavior control software.

**MF:** As Miles said, we’re both involved in a wide range of projects. One that I’ve been particularly focused on over the past few years, and continue to work on, is the development and maintenance of several data visualization websites for the IBL. These platforms are designed to showcase the datasets we’ve collected and make them accessible through interactive, intuitive interfaces. They serve two main audiences, newcomers who want to get a broad sense of the scope and structure of the data and IBL scientists who need to explore the details and nuances of their experiments more deeply. The goal is to make complex data more approachable, explorable, and ultimately more useful for a range of users.

### The IBL aims to build a model of how brains make decisions. Software development involves a lot of decision-making, and research software is often an aid to help researchers decipher data and make “decisions.” Does this sound like a strained comparison? Do you see overlap between the research goals of the IBL and the work you do as a software engineer?

**MF:** There are definitely overlaps between the research goals of the IBL and the work that we do. The infrastructure, algorithms, and tools that we develop as software engineers allow the scientists to navigate the complex data and test scientific hypotheses, in effect making “decisions.”

**MW:** Indeed, everything we do as humans centers around decisions; however, humans operate very differently to computer programs—namely, we have an ability to learn and adapt to changes in our environment. Perhaps one day the decisions we make developing software tools can be taken by a computer, learning from the demands of the users. It’s still broadly true that humans are much better than computers at performing certain tasks and vice versa. I’m often reminded of this when developing software, and hopefully the IBL will bring us closer to understanding why.

### Do you think humanity in general will benefit from a better understanding of how our brains make decisions?

**MW:** Yes, humans as a species are very successful, yet as individuals we don’t always make statistically optimal decisions. Understanding where this diversity of thought arises and how it helps us navigate the world will hopefully allow us to better appreciate what makes us thrive on both an individual and population level.

**MF:** I agree with Miles, and I also think we can benefit when this knowledge allows us to further our understanding of mental health or neurological disorders. An example of such a project in the IBL is comparing the decision-making processes in so-called “healthy” adult brains to those with autism. We can start to understand how autistic brains might approach decisions differently and get a more nuanced view of what neurodiversity really means.

### The software developed by the IBL is open for anyone to use. How often do you receive input or code contributors from people outside the IBL?

**MW:** As a leader in outreach, Mayo can probably give a better response than me, but we certainly receive a lot of valuable feedback and feature requests from external users of our tools. That said, we don’t often receive code contributions. I’m not sure if this is a reflection of the community or of our codebase!

**MF:** Yes, we mainly get useful feedback or feature requests from users outside the IBL who want to use our code in their research. There are a few examples where our code has been incorporated into other workflows and expanded upon, but on the whole the software developers at the IBL are the main contributors.

### What do you see as the biggest advantages of open-source development?

**MF:** For me, one of the biggest advantages of open-source practices is that they democratize access to tools and data. By removing cost and privilege barriers, open source allows a much broader and more diverse group of people to participate in scientific research and software development. Another advantage is that it promotes transparency and reproducibility. When the code and data are openly available, it’s easier to understand and reproduce technical and scientific results, which strengthens the research.

**MW:** Yes, I think we in the field of neurophysiology were much slower to develop community tools for data acquisition, processing, and analysis. This is quickly changing, and I think it is having a huge impact on reproducibility.

### Are there disadvantages as well?

**MW:** Many of the tools we use are highly niche, which can present challenges to keeping software projects alive as the community will always be small. Many of the tools we work with were created by a researcher during a research project, and they simply don’t have the time or motivation to maintain and improve the code—they’d rather be doing the research. This is where funding for permanent staff is a boon not just for individual labs or projects but for the community at large.

**MF:** Yes, there are many open-source projects that are closely tied to specific funding cycles or rely heavily on the efforts of just one person or a small team. When those individuals move on to new roles or projects, the tools they created often become unsupported or fall out of date.

### The “research software engineer” job role is relatively new. Has this role gained enough traction that you see a long-term career path as a research software engineer, or do you see it as a stepping stone to something else? Are you committed to staying in open-source development?

**MF:** It’s been really encouraging to see how much traction the research software engineer role has gained over the five years I’ve been working at the IBL. It’s increasingly recognized as a vital part of scientific research, especially as advances in technology have made it possible to collect massive datasets. We often hold the technical knowledge that bridges the gap between scientific goals and practical implementation, and I feel this is being recognized by the community. There are more and more research centers that have funding specific for research software engineer roles, and this has made it feel like a more viable long-term career path. I’m committed to staying in open-source development, it aligns with my values around collaboration, transparency, and making science more accessible.

**MW:** It’s not only encouraging to see more of these roles appear but also since I started my career in research, I have witnessed a huge shift in the adoption of open data and community tools. That said, there are still very few such positions and fewer still have long-term funding. In my experience, there is little or no support for career progression and lower pay than in industry.

### What do you enjoy most about working at the IBL in your current roles?

**MF**: What I enjoy most about my current role is the variety of work it offers. While each of us tends to specialize in a particular area, the small size of our team means that we all need to stay informed about the different ongoing projects. This creates an environment where continuous learning is essential, and we regularly need to develop new skills across different contexts.

**MW:** I too enjoy learning new skills on the job. Not only is there a diverse range of work, but we are also able to take the time to thoroughly understand and optimize the areas that interest us most, as the code we write is very often applicable to several projects. I especially enjoy working on tools that have a well-defined scope and are useful to the wider community. It’s certainly gratifying when someone naturally encounters your work and finds it valuable.

